# 

**Published:** 1889-12-21

**Authors:** 


### NOTICE TO CORRESPONDENTS.

All MS., Letters, Books for Review, and other matters intended for the Editor, should be addressed The Editor, The Lodge, Porchester Square, London, W.

**Notice to Advertisers and Subscribers.**

All Advertisements, Orders for Copies of The Hospital, and Business Communications should be addressed to The Publisher, *not to the Editor*, The Hospital, 139-140, Salisbury-court, Fleet-street, E.C.

**Bound Volumes of "The Hospital."**

Vol. VI., containing full page portraits of T.R.H. the Prince and Princess of Wales, and Miss Florence Nightingale, is now ready, 4s., post free. Cases for binding, may be had of the Publisher, at 1s. 6d. each.

**To Residents, Secretaries, and Matrons**

The Editor will be glad to have the Regulations and each Report as issued by Asylums, Hospitals, Convalescent and Nursing Institutions, as well as those of other Charities, and an early copy in duplicate of all Appeals and Festival Notices, etc., addressed to *The Editor, The Lodge, Porchester-square, London, W.* Any superintendent, secretary, matron, or other chief official who regularly sends such information may be registered, on application to the Publisher, to receive free of cost a cover for binding The Hospital in half-yearly volumes.

### The Student's Column.

### UNIVERSITY OF LONDON.

M.B. EXAMINATION, OCT., 1889. EXAMINATION FOR HONOURS.

Medicine.—*First Class.*—C. MacG. Kitching (*Gold Medal*), Guy's; Ransom Pickard (*Gold Medal*), St. Bartholomew's; William Soltau Fenwick, London Hospital and Strassburg and Berlin; Milton Prentice Ledward, Owens College and Manchester Royal Infirmary; John Edward Platt, Owens College and Manchester Royal Infirmary. *Second Class.*—John Arthur Hayward, St. Bartholomew's; Henry Moore Bowman, St. Bartholomew's; Percy James Duncan, Charing Cross; Robert Devereux Mothersole, Guy's; Charles Frederick Seville, Owens College and Manchester Royal Infirmary; Edgar Olive Turner, University College; Charles Pardey Lukis, St. Bartholomew's. *Third Class.*—Edward Percy Paton, St. Bartholomew's; Ernest Hugh Snell, *B.Sc.*, Queen's College, Birmingham; Joseph Johnston Macgregor, St. Bartholomew's.

Obstetric Medicine.—*First Class.*—Edward Percy Paton (*Scholarship and Gold Medal*), St. Bartholomew's. *Second Class.*—Ransom Pickard, St. Bartholomew's; George Henry Barker, *B.Sc.*, Bristol Medical School; Charles MacGowan Kitching, Guy's; John Arthur Hayward, St. Bartholomew's; Charles Frederick Seville, Owens College and Manchester Royal Infirmary. *Third Class.*—Joseph Johnston Macgregor, St. Bartholomew's; William Soltau Fenwick, London Hospital and Strassburg and Berlin; Robert Devereux Mothersole, Guy's.

Forensic Medicine.—*First Class.*—Henry Moore Bowman (*Scholarship and Gold Medal*), St. Bartholomew's; John Edward Platt (*Gold Medal*), Owens College and Manchester Royal Infirmary; Joseph Johnston Macgregor, St. Bartholomew's; Ransom Pickard, St. Bartholomew's; Henry Johnstone Campbell, Guy's. *Second Class.*—Richard Oxley Bowman, Owens College and Manchester Royal Infirmary; Mary A. McCulloch Knight, London School of Medicine for Women; Walter Henry Brazil, Owens College. *Third Class.*—Ernest Hugh Snell, Queen's College, Birmingham; Edward Percy Paton, St. Bartholomew's; John Price Williams, Owens College and Manchester Royal Infirmary; Charles Frederick Seville, Owens College and Manchester Royal Infirmary; Robert Devereux Mothersole, Guy's.

M.D. EXAMINATION.

Pass List.—*Medicine.*—John Hill Abram, Evelyn Oliver Ashe, Percy Ashworth, *B.S.*, *B.Sc.*, James Thomas Bays, Robert Bird, *B.S.*, J. R. Bradford, *D.Sc.*, Gold Medal, Weldon Cragg Carter, Wayland Charles Chaffey, William Frederick Clarke, *B.S.*, Herbert Evelyn Crook, *B.S.*, Hilarn. M. Fernando, *B.Sc.*, John Lacy Firth, Theodore Fisher, Leonard Maurice Gabriel, John Edwin Gould, George William Hill, *B.S.*, Arthur Pearson Luff, *B.Sc.*, Henry Grabham Lys, Henry John Macevoy, *B.Sc.*, Alfred Alexander Mumford, C. Drummond Muspratt, *B.S.*, John Porter Parkinson, William Bramwell Ransom, *B.Sc.*, William Newton Risdon, *B.S.*, Harry Arthur Sansom, Hugh Smith, Edgar Herbert Thane, Edward Francis Willoughby, William George Willoughby.

*State Medicine.*—Charles Edward Baddeley, F. François Burghard, *M.S.*, William Barltrop Featherstone.

B.S. EXAMINATION.

Pass List.—*First Division.*—Alfred George Francis, Charles Arthur Kent, *B.Sc.*, Charles MacGowan Kitching, Robert Devereux Mothersole, Berkeley George A. Moynihan, Gilbert Benjamin Mower White.

*Second Division.*—George Black, William Soltau Fenwick, Alfred Downing Fripp, Francis Horatio Napier.

M.S. EXAMINATION.

Pass List.—Henry Percy Dean, *B.Sc.*, Alfred Parkin.

### Scraps and Gleanings.

The death is announced of Mr. Charles Irving, surgeon, at Tunbridge.

Dawlish Dispensary treated 275 cases last year; the expenses were £90.

The Queen is dispensing £300 to the poor of Windsor as a New Year's gift.

Several districts of Russia, in the Volga region, are devastated by famine.

Dr. Creighton has been examined by the Royal Commission on Vaccination.

Nineteen gentlemen have subscribed £100 each to Ayr County Hospital to reduce the debt.

Princess Beatrice presented the certificates gained by ambulance pupils at Windsor last week.

Deputy-Surgeon General J. Landale has been appointed principal medical officer on the staff at Cork.

Connecticut Hospital Society has received a bequest of £10,000 from the late Mrs. Ellen M. Gifford.

Influenza is spreading everywhere: in London and New York hundreds of people are suffering from it.

Mr. Charles D. Holmes' workmen at Hull have all given quarter of a day's pay to the infirmary; it amounts to £49.

The Surgical Aid Society helped 8,747 patients last year, a number considerably larger than they have ever helped before.

Mr. T. H. Oppenshaw has been appointed surgical registrar to the London Hospital in the place of Mr. J. Hutchinson, jun.

Mrs. Trayner took the chair at the annual meeting of Edinburgh Hospital and Dispensary for Women. A deficit of £24 was reported.

Miss Barrow, matron of Chester Infirmary, will be glad of toys or money to help the usual Christmas entertainments for the patients.

A "Mary Stanley" bed has been established in the Hospital of St. John of Jerusalem and St. Elizabeth of Hungary, Great Ormond-street.

The health of Ceylon has not been satisfactory of late, fevers of a malarial type and cholera having prevailed in several parts of the island.

Newcastle-on-Tyne Hospital for Diseases of Women is to have a separate building of its own. For this purpose a committee has been formed.

The public school at New Somerby, Grantham, has been closed on account of an epidemic of influenza which has broken out among the children.

The Duke of Westminster has sent £500 to the Chester Infirmary, this money having been paid during the year by visitors desiring to see Eaton Hall.

At Plumstead 270 cases of insanitary houses are reported. Many of them were in a shocking state, destitute of sanitary appliances, and built on a ditch.

Birmingham Dental Hospital treated 6,781 patients last year. In 530 cases anæsthetics were given. Alas! how we poor mortals suffer from toothache.

The Houses of Parliament are now to be thoroughly lighted by electricity, the work being placed in the hands of the Westminster Electric Supply Company.

Miss H. Connor's death is reported. She was house surgeon of the Lady Aitchison Hospital for Women, Lahore, having obtained her diploma and certificate last spring.

Sir Reginald Hansen presided at the annual dinner in connection with the Infant Orphan Asylum at Wanstead. Subscriptions were announced to the amount of £2,000.

Mr. C. Leopold Hudson, F.R.C.S.Eng., has been appointed to the office of surgical registrar of the Middlesex Hospital, vacant by the resignation of Mr. W. Roger Williams.

Cumberland Hospital Sunday Fund has been awarded as follows: Cumberland Infirmary, £555; Carlisle Dispensary, £51; Carlisle House of Recovery, £97; Workington Infirmary, £135.

Worcester Dispensary proposes to take another surgeon on the staff—making nine in all. Dr. Woodward, who has been connected 30 years with the institution, is now merely an honorary officer.

Dr. Augustus Voelcker, chemist to the Royal Agricultural Society, has been commissioned by Lord Cross to visit India and study on the spot the best scientific methods for improving Indian agriculture.

Hastings Hospital Sunday Fund has been awarded as follows: Hospital, £451; Homœopathic Dispensary, £47; Buchanan Hospital, £114; Free Dispensary, £80; Provident Dispensary, £57; Bean Site Home, £93.

Sir Benjamin Samuel Phillips, late of Grosvenor-street, London, left by his will £100 each to the London Hospital, the Royal Hospital for Incurables, Sussex County Hospital, the Farringdon Dispensary, and the Metropolitan Hospital.

The late Mr. S. Fielden, of Centre Vale, Todmorden, has taken steps towards providing a hospital for infectious diseases at Sourhall for the whole of the Todmorden union and the borough of Bacup. The plans are already in a forward condition.

The council of the Royal College of Surgeons in Ireland have resolved to confer the honorary fellowship of the college on Mr. Thomas H. Parke, in recognition of his distinguished services to the members of Mr. Stanley's Emin Pasha Relief Expedition.

The opening ceremony of the St. Leonard's Hospital Bazaar was a very grand affair. Archdeacon Sutton offered a prayer, and then the Mayor welcomed the Duchess of Cleveland. Lord Brassey and Col. Brookfield were among the speakers, and the volunteers furnished a guard of honour.

The gross value of the personal estate of the late Mr. William Westgarth has been sworn at £147,289, and the net value at £145,825. He bequeaths £100 to the Bethnal Green Free Library, £500 each to the Beaumont Institute, the Edinburgh Infirmary, the Young Men's Christian Association, and the Young Women's Christian Association.

